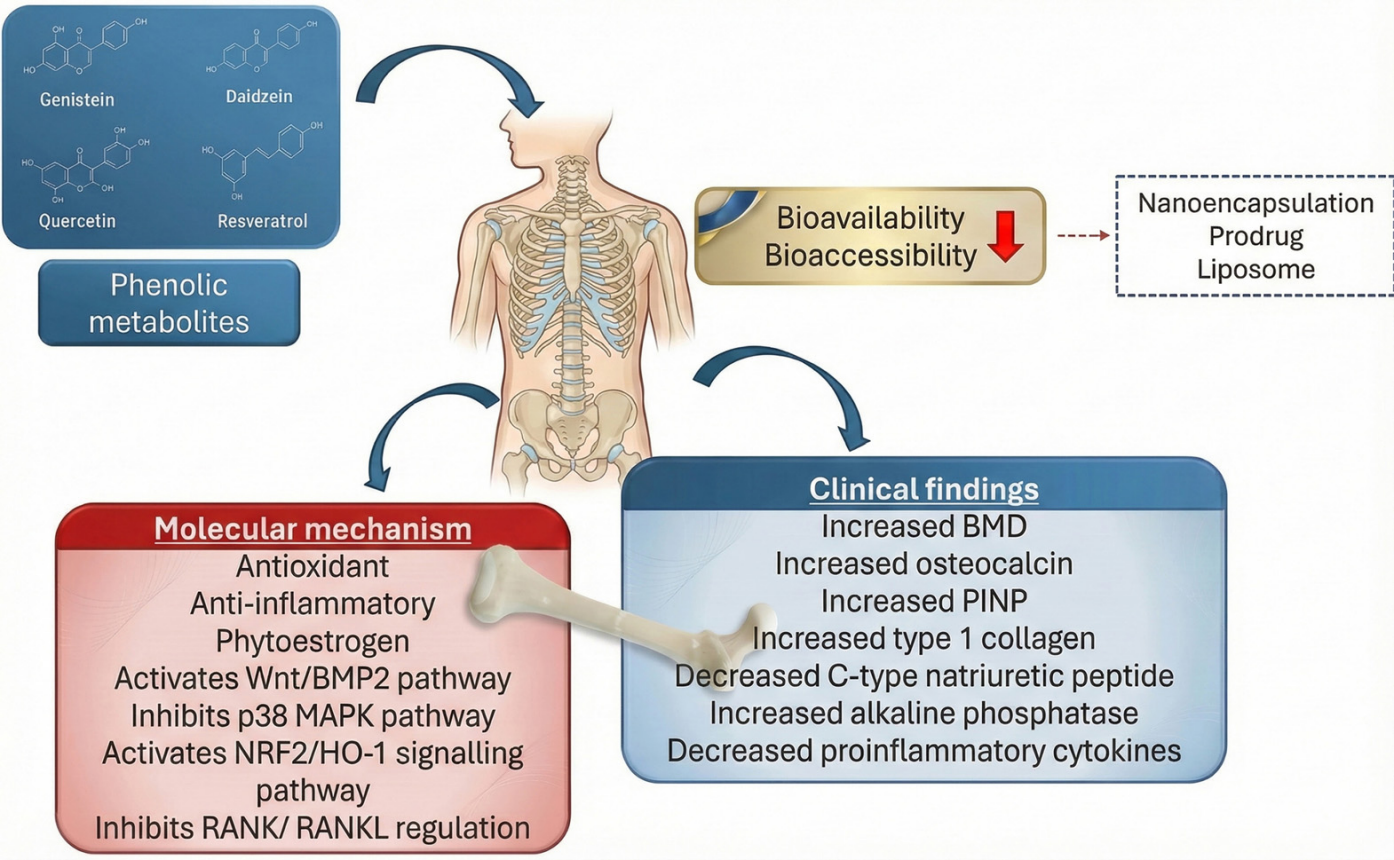# Phenolic-derived compounds in osteoporosis—Mechanisms, clinical evidence, and drug delivery: A review

**DOI:** 10.17305/bb.2025.13301

**Published:** 2025-12-19

**Authors:** Haryati Ahmad Hairi, Rusdiah Ruzanna Jusoh, Muhammad Zulfiqah Sadikan, Ahmad Nazrun Shuid

**Affiliations:** 1 Department of Biochemistry, Faculty of Medicine, Manipal University College Malaysia, Melaka, Malaysia; 2 Faculty of Pharmacy and Health Sciences, Universiti Kuala Lumpur Royal College of Medicine Perak, Ipoh, Perak, Malaysia; 3 Department of Pharmacology, Faculty of Medicine, Universiti Teknologi MARA (UITM), Sungai Buloh, Selangor, Malaysia

**Keywords:** Osteoporosis, bone remodeling, phenolic compounds, clinical findings, bioavailability, drug delivery system

## Abstract

Osteoporosis is a degenerative skeletal disorder characterized by reduced bone mass and the deterioration of bone microarchitecture, resulting in an increased risk of fractures. Its development is driven by an imbalance in bone remodeling, where osteoclastic bone resorption surpasses osteoblastic bone formation. Factors such as oxidative stress, chronic inflammation, ferroptosis, and hormonal changes, particularly estrogen deficiency in postmenopausal women, contribute to this imbalance. Metabolites derived from phenolic compounds have emerged as promising natural agents for osteoporosis prevention due to their antioxidant, anti-inflammatory, and hormone-modulating properties. Key phenolic groups, including flavonoids (quercetin), isoflavones (genistein and daidzein), and stilbenes (resveratrol), have demonstrated significant osteoprotective effects by regulating receptor activator of nuclear factor-kappa B ligand (RANKL) and osteoprotegerin (OPG) signaling, activating Wnt and β-catenin pathways, and suppressing inflammatory cytokines. Clinical findings indicate that these compounds may enhance bone mineral density and modulate bone turnover markers in populations at risk for osteoporosis. However, their clinical application is limited by low bioavailability and rapid metabolism. Advances in drug delivery systems, including nanoencapsulation, liposomal formulations, and prodrug design, have improved stability, absorption, and targeted delivery to bone, thereby enhancing therapeutic potential while minimizing systemic effects. This review discusses the molecular mechanisms underlying osteoporosis, emphasizing oxidative and hormonal dysregulation, and highlights the therapeutic relevance of phenolic compounds. Additionally, it summarizes recent clinical observations and formulation strategies aimed at enhancing therapeutic efficacy. Overall, phenolic compounds represent promising plant-based strategies for the prevention and management of osteoporosis.

## Introduction

Phenolic compound-derived secondary metabolites, a significant subclass of plant-derived natural products, are structurally diverse, low-molecular-weight organic compounds (typically <3000 Da) synthesized as part of the plant’s intrinsic defense mechanisms. These bioactive molecules play a critical role in a plant’s ability to respond to environmental challenges through their antioxidant, antimicrobial, and signaling properties. By acting as protective agents, they enhance stress resilience, facilitate adaptive responses, and promote survival under adverse conditions. From an evolutionary perspective, secondary metabolites have emerged as essential regulators of plant defense, a central theme explored in this review concerning their structural complexity and diverse biological functions [1]. Beyond their ecological significance, these compounds possess broad therapeutic potential in human health, with applications in the treatment of chronic conditions such as diabetes [2], neurodegenerative diseases [3], various cancers [4], and notably, osteoporosis [5]. Their pharmacological efficacy is primarily attributed to their ability to mitigate oxidative stress, preserve cellular function, and modulate disease-related pathways. Within this context, phenolic compounds—including flavonoids, phenolic acids, stilbenes, and lignans—have garnered considerable attention for their role in bone health. Increasing clinical and experimental evidence supports their use in the prevention and management of osteoporosis, acting through multiple mechanisms to support bone remodeling, reduce bone resorption, and protect against oxidative damage-induced bone loss [6].

Osteoporosis is a significant global public health concern, affecting millions of people worldwide. It is characterized by decreased bone mass and the deterioration of bone microarchitecture, changes that are not merely a normal consequence of aging. These alterations substantially increase the risk of fractures, particularly in the hip, spine, and wrist, leading to disability, diminished quality of life, and higher mortality rates. The socioeconomic impact of osteoporosis is considerable, contributing to rising healthcare costs and prolonged medical care dependency among affected individuals. Fractures represent the most severe complication of osteoporosis, with their incidence rising sharply with age. Globally, approximately one in three women is at risk of sustaining an osteoporotic fracture, accounting for 20% to 25% of all individuals who will experience such a fracture during their lifetime. Among those suffering from osteoporosis-related hip fractures, mortality rates within the first two years range from 12% to 20% [7]. Furthermore, nearly half of older adults become dependent on others for care following a fracture, underscoring the urgent need for effective prevention strategies [8].

Numerous therapeutic strategies have been explored to address osteoporosis; however, multi-target drug ligands demonstrate superior efficacy in managing this multifactorial and complex disease compared to single-target agents or combination therapies. In recent years, significant advancements have been made in osteoporosis treatment through the application of medicinal plants. Clinical trials increasingly support the beneficial role of medicinal plants and their bioactive secondary metabolites in attenuating bone loss and improving skeletal health. Complementary *in vitro* and *in vivo* studies have further elucidated the positive effects of these compounds on bone metabolism. Despite these advances, gaps remain in the comprehensive understanding of the chemical diversity, bioactive profiles, and full spectrum of molecular mechanisms through which these metabolites exert osteoprotective effects. Additionally, limitations related to bioavailability, metabolic stability, and clinical translation continue to pose challenges. This review aims to critically evaluate the therapeutic potential of plant-derived secondary metabolites, specifically phenolic compounds, in the prevention and management of osteoporosis. Emphasis is placed on their molecular mechanisms of action, which involve enhancing osteoblastogenesis, inhibiting osteoclast activity, and modulating key signaling pathways. Additionally, clinical findings, challenges in pharmacokinetics (absorption and metabolism), and current strategies to enhance bioavailability—including advanced drug delivery systems—are discussed to provide a comprehensive perspective on their application in anti-osteoporosis therapy.

## Phenolic compounds

**Figure 1.**
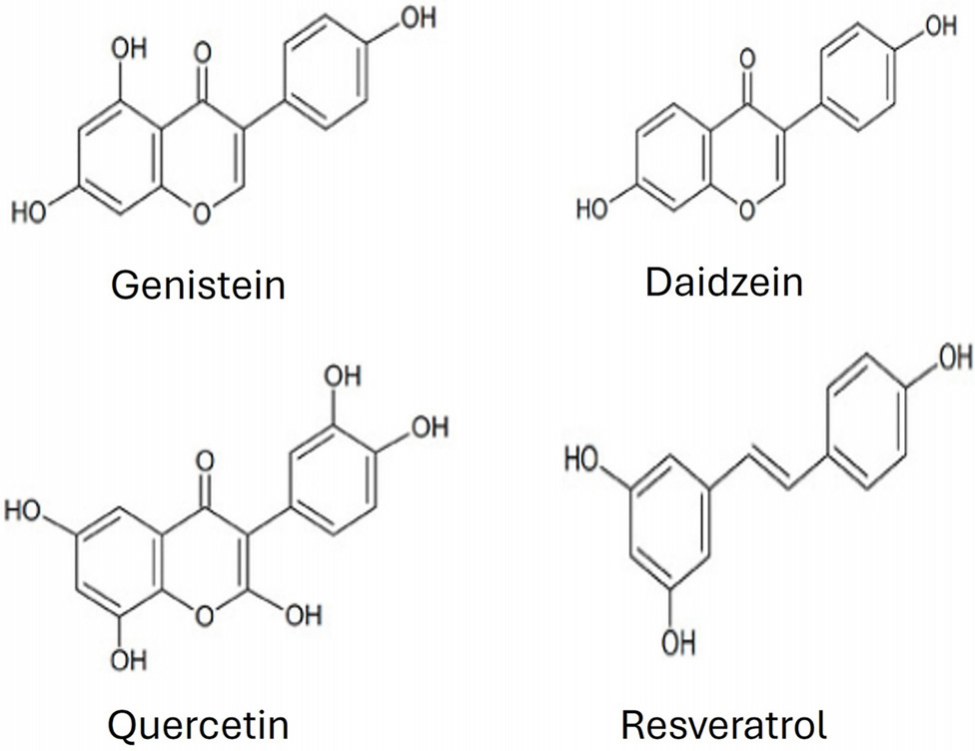
**Chemical structure of phenolic compounds.** This figure illustrates the chemical structures of the isoflavones genistein and daidzein, the flavonol quercetin, and the stilbene resveratrol (3,4′,5-trihydroxy-trans-stilbene).

Phenolic compounds are characterized by the presence of at least one aromatic ring and one or more hydroxyl groups. This group encompasses a diverse range of molecules, including simple phenols, polyphenols, stilbenes, and lignans (Figure 1). In plants, they contribute to cell wall architecture by forming cross-links with macromolecules such as cellulose, hemicellulose, and pectin, thereby reinforcing the structural integrity and compactness of the cell wall matrix [9]. Many phenolic compounds, particularly flavonoids, exhibit potent antioxidant and free radical scavenging activities. Others, such as genistein and daidzein, possess phytoestrogenic properties [10].

Simple phenols represent the most basic form of phenolic compounds, characterized by one or more hydroxyl groups (–OH) attached to a single aromatic ring (C6). Common examples include hydroquinone, catechol, and pyrogallol. Although structurally simple, these compounds possess significant biological activities, including antioxidant, antimicrobial, and anti-inflammatory effects [11]. Polyphenols are complex compounds distinguished by the presence of two or more phenolic rings. This diverse group, including flavonoids, phenolic acids, and tannins, is commonly found in plant-derived foods and beverages. Polyphenols often exist in conjugated forms, covalently bonded to one or more sugar units, typically through O-glycosidic linkages, although C-glycosidic bonds are also observed, albeit less frequently. Furthermore, polyphenols can form ester linkages with organic acids, as seen in compounds like chlorogenic acids and catechins found in green tea [12]. Polyphenolic compounds are widely recognized for their broad spectrum of health-promoting effects, including a reduced risk of chronic conditions such as cancer, cardiovascular disease, diabetes, osteoporosis, and neurodegenerative disorders. These bioactivities are largely attributed to their potent antioxidant, anti-inflammatory, anticancer, antimicrobial, antidiabetic, and antihypertensive properties. Among these, the antioxidant capacity of polyphenols is considered a central mechanism underlying their protective effects, particularly against oxidative stress, a key contributor to cellular damage and the pathogenesis of chronic diseases [13].

Polyphenols exert their antioxidant effects through multiple pathways, including the direct scavenging of reactive oxygen species (ROS), chelation of pro-oxidant transition metal ions, and inhibition of oxidative stress-associated enzymes, such as xanthine oxidase and NADPH oxidase. These mechanisms collectively contribute to maintaining redox homeostasis and preventing oxidative damage to lipids, proteins, and DNA. Epidemiological and clinical studies consistently demonstrate that long-term dietary intake of polyphenol-rich foods is associated with improved health outcomes and a lower incidence of age-related degenerative diseases [14]. Their multifunctional roles underscore the therapeutic potential of polyphenols as dietary agents or adjunctive treatments in managing complex diseases, including osteoporosis.

Stilbenes represent a significant subclass of non-flavonoid polyphenols, characterized by a core 14-carbon structure consisting of two benzene rings connected by an ethylene bridge. The central ethylene group linking the aromatic rings is crucial for their structural and functional properties [15]. Resveratrol (3,4′,5-trihydroxy-trans-stilbene) is one of the most extensively studied stilbenes, found naturally in grapes, red wine, peanuts, chocolate, and mulberries. It has been reported to exhibit a wide range of beneficial properties, including antioxidant, anti-inflammatory, anti-platelet, anticancer, and anti-osteoporotic effects [16].

Lignans are stereospecific dimers formed through the bonding of cinnamic alcohols (monolignols) at the carbon 8 (C8–C8) position. These bioactive, non-nutritive phenolic constituents of plant-based foods contribute minimally to caloric intake while exhibiting significant physiological and health-promoting effects. Lignans are most concentrated in flax and sesame seeds but are also present in smaller quantities in grains, other seeds, fruits, and vegetables. Within plants, lignans typically occur either in their free form or bound to sugars [17]. They are categorized into seven primary types: secoisolariciresinol (Seco), pinoresinol (Pino), matairesinol (Mat), medioresinol (Med), sesamin (Ses), syringaresinol (Syr), and lariciresinol (Lari). Notably, many lignans possess therapeutic properties, including antioxidant, anticancer, anti-inflammatory, antibacterial, and antifungal effects [18]. Specifically, lignans are known to exert antioxidant and anti-inflammatory activities and modulate pathways dependent on estrogen receptors (ERs). Due to these properties, lignans hold potential as therapeutic agents for managing postmenopausal symptoms, such as cardiovascular disease and osteoporosis. The molecular mechanisms underlying lignans’ effects in these diseases involve the inhibition of inflammatory signaling pathways, particularly the nuclear factor (NF)-κB pathway [19].

## Mechanisms of osteoporosis pathogenesis

Osteoporosis is a prevalent skeletal pathology characterized by reduced bone mineral density (BMD) and compromised microarchitectural integrity, which collectively exacerbate fracture susceptibility and contribute significantly to the global disease burden. Clinically, osteoporosis is defined by a BMD T-score of ≤ −2.5, indicating a reduction of 2.5 standard deviations or more below the young adult mean reference [20]. Despite this clear diagnostic criterion, osteoporosis remains markedly underdiagnosed, with its epidemiological prevalence often inferred indirectly from fracture incidence data.

Under normal physiological conditions, bone remodeling maintains a dynamic equilibrium between osteoblastic bone formation and osteoclastic bone resorption. In the pathogenesis of osteoporosis, this equilibrium is disrupted, favoring resorption over formation, which leads to net bone loss and degradation of trabecular and cortical microarchitecture. The condition predominantly affects elderly populations, particularly postmenopausal women, where estrogen deficiency, comorbid chronic conditions, and prolonged exposure to specific pharmacotherapies (including glucocorticoids) act as critical etiological factors [21]. The fundamental molecular mechanism driving osteoporosis is the disruption of the homeostatic balance between bone resorption and formation, arising from functional abnormalities or altered cellular populations of osteoblasts and osteoclasts, resulting in excessive bone degradation relative to bone synthesis.

### Bone remodeling imbalance

Bone homeostasis is primarily maintained through the dynamic equilibrium between osteoclast-mediated bone resorption and osteoblast-mediated bone formation. Throughout life, bone tissue undergoes continuous remodeling, a tightly regulated process involving the coordinated activity of bone marrow-derived mesenchymal stem cells (BMMSCs), osteoblasts, osteocytes, and osteoclasts [22]. BMMSCs possess multipotent differentiation potential, giving rise to osteogenic, adipogenic, and chondrogenic lineages. They contribute to bone formation by promoting osteogenesis and facilitating calcium deposition [23].

Osteoblasts, which originate from BMMSCs, are key effectors in the bone formation process. Primarily located on bone surfaces, they synthesize the extracellular bone matrix through the secretion of type I collagen (Col1) and various bone matrix proteins. In addition to collagen, osteoblasts secrete essential components required for bone mineralisation, including chondroitin sulphate, inorganic phosphate, and calcium ions, thereby playing a critical role in matrix maturation and mineral deposition [24]. Osteoclasts are specialized, terminally differentiated cells derived from the hematopoietic monocyte-macrophage lineage. These multinucleated cells play a vital role in bone remodeling by resorbing mineralized bone matrix through the secretion of organic acids and proteolytic enzymes, which dissolve the mineral and organic components of bone, thereby maintaining skeletal structure and function [25]. Osteocytes, originating from osteoblasts and embedded within the mineralized bone matrix, serve as key regulators of bone homeostasis. They function as mechanosensors and orchestrators of bone remodeling by detecting mechanical and biochemical signals and modulating the release of cytokines and signaling molecules that influence osteoclast and osteoblast activities [26].

However, this tightly regulated balance is disrupted under pathological conditions. Hyperactivation of osteoclasts, coupled with impaired or diminished osteoblastic activity, leads to excessive bone resorption and inadequate bone formation. This imbalance ultimately compromises skeletal integrity, promoting the progression of osteoporosis over time [27]. During bone homeostasis and repair, several key signaling pathways regulate skeletal development, remodeling, and regeneration. Among these, the Wnt/β-catenin, bone morphogenetic protein (BMP-2)/Smad, and phosphatidylinositol-3-kinase/protein kinase B (PI3K/Akt) pathways play central roles in orchestrating bone growth and cellular differentiation.

Wnt signaling serves as a crucial regulatory pathway directing the lineage-specific differentiation of mesenchymal stem cells (MSCs) into osteoblasts, thereby playing a central role in bone formation, development, and remodeling. This osteogenic differentiation is achieved by inhibiting adipogenic transcription factors, such as PPARγ, while concurrently activating osteogenic transcription factors, including Runx2 and osterix. By modulating the transcriptional landscape of bone marrow progenitor cells, Wnt signaling effectively shifts cellular commitment from adipogenesis to osteogenesis, thus enhancing bone formation and skeletal integrity [28]. This regulatory mechanism is vital for maintaining the balance between bone and fat formation within the bone marrow microenvironment, a balance often disrupted in aging and osteoporosis. The signaling pathway is mediated by the Wnt family, which comprises 19 secreted glycoproteins known to regulate key cellular processes such as proliferation, differentiation, and apoptosis. These Wnt proteins initiate intracellular signaling cascades that further fine-tune the functional behaviour of osteoblast precursors, reinforcing the pathway’s integral role in skeletal biology [29].

Additionally, the Wnt signaling pathway plays a crucial role in maintaining the dynamic equilibrium between osteoblast-mediated bone formation and osteoclast-mediated bone resorption, both fundamental processes of physiological bone remodeling. This signaling network operates through two primary branches: the canonical (β-catenin-dependent) and non-canonical (β-catenin-independent) pathways, which contribute distinct yet complementary functions to skeletal homeostasis. Disruption or dysregulation in either branch impairs the tightly coordinated interplay between osteoblasts and osteoclasts, ultimately compromising bone remodeling and increasing susceptibility to skeletal pathologies, including osteoporosis [30]. A clear illustration of this regulatory complexity is provided by Wnt3a, a key ligand within the Wnt signaling family that modulates bone remodeling predominantly via the canonical Wnt/β-catenin pathway. In osteoporotic models, activation of Wnt3a results in increased secretion of osteoprotegerin (OPG), a decoy receptor that binds to receptor activator of NF-κB ligand (RANKL). By preventing the RANK–RANKL interaction essential for osteoclast differentiation and activation, Wnt3a indirectly suppresses osteoclastogenesis. This mechanism promotes a favorable osteoblast-to-osteoclast ratio, thereby accelerating bone repair and enhancing skeletal integrity [31]. Conversely, silencing Wnt3a expression leads to impaired osteoblast differentiation and reduced matrix mineralization, underscoring its essential role in osteogenesis [32]. In addition to its anabolic effects, Wnt3a directly inhibits osteoclast differentiation by acting on bone marrow-derived monocyte-macrophage lineage cells (BMMs) through the canonical Wnt signaling pathway [33]. Similarly, Wnt1 has been shown to suppress osteoclastogenesis *in vitro* via canonical Wnt signaling in RAW264.7 cells, a murine monocyte–macrophage leukemia cell line, further reinforcing the anti-resorptive function of Wnt ligands in skeletal homeostasis [34].

Bone morphogenetic protein 2 (BMP2) is a potent growth factor that stimulates osteoblast and osteoclast activity, playing a central role in osteoblast differentiation and bone formation through the BMP2/SMAD signaling pathway. As a member of the transforming growth factor-β (TGF-β) superfamily, BMP2 is essential for bone development. It promotes the differentiation of MSCs into osteoblasts, thereby contributing to the prevention of bone diseases and fractures. BMP2 exerts its effects by binding to type I BMP receptors (BMPR-1) on the cell membrane, initiating the phosphorylation of intracellular signaling proteins Smad1 and Smad5. Once phosphorylated, Smad1/5 forms a complex with Smad4, which translocates from the cytoplasm to the nucleus. In the nucleus, this complex activates the transcription of critical osteogenic genes, including runt-related transcription factor 2 (Runx2) and osterix (Osx), thereby promoting the osteogenic differentiation of BMMSCs [35, 36]. Runx2, a downstream target of BMP2/Smad signaling, is essential for osteoblast differentiation, enhancing the transcription of genes involved in chondrocyte maturation and mineralization, such as osteocalcin (OCN), Col1, and alkaline phosphatase (ALP)—key markers of mature osteoblasts. Osx, a zinc finger transcription factor acting downstream of Runx2, further supports osteogenic differentiation by regulating the expression of late-stage osteoblast-specific genes and functional proteins [36].

The phosphatidylinositol 3-kinase/protein kinase B (PI3K/AKT) signaling pathway is vital in regulating osteoblast proliferation, survival, and differentiation. PI3K, an enzyme that catalyzes the production of the second messenger phosphatidylinositol 3,4,5-trisphosphate (PIP3), initiates a cascade of downstream signaling events. Meanwhile, AKT, a key effector downstream of PI3K, becomes activated through phosphorylation and promotes cell proliferation, survival, and differentiation. Previous studies have demonstrated that activating the PI3K/AKT pathway enhances osteoblast proliferation and differentiation, thereby accelerating bone formation. This pathway upregulates the expression of key osteogenic genes, including ALP, OCN, and bone matrix proteins, all essential for osteoblast maturation and bone matrix synthesis [37]. Furthermore, activation of this signaling cascade enhances calcium ion transport and the mineralization capacity of osteoblasts [38]. Given its central role in osteoblast differentiation and function, the PI3K/AKT signaling pathway represents a promising therapeutic target for treating hormone-induced osteoporosis.

Osteoclastogenesis is primarily regulated by a network involving RANKL, its receptor RANK, OPG, and monocyte colony-stimulating factor (M-CSF). Osteoblasts secrete RANKL, which binds to RANK on osteoclast precursors to promote their recruitment and initiate the bone remodeling process. This RANKL–RANK interaction enhances osteoclast differentiation, activity, and survival. In contrast, OPG acts as a decoy receptor by binding to RANKL and preventing its interaction with RANK, thereby inhibiting osteoclast proliferation [39]. Notably, the binding of RANKL to RANK also triggers the release of pro-inflammatory cytokines, such as TNF-α, IL-1, and IL-7, while activating key transcription factors, including c-Fos and NFATc1, which drive osteoclast differentiation [40]. The balance between RANKL and OPG, alongside the communication between osteoblasts and osteoclasts, plays a crucial role in regulating bone turnover and remodeling. When bone resorption surpasses bone formation, this imbalance leads to bone loss and contributes to the development of osteoporosis.

This delicate balance between bone formation and resorption can be disrupted by various internal and external factors, including oxidative stress, ferroptosis (a form of iron-dependent cell death), estrogen deficiency, aging, and chronic inflammation. These pathological conditions can impair osteoblast function, enhance osteoclast activity, or interfere with critical signaling pathways, such as the Wnt/β-catenin, BMP-2/Smad, and PI3K/Akt pathways, which regulate bone remodeling. Due to this regulatory imbalance, bone resorption may exceed bone formation, resulting in reduced bone density and structural degradation, hallmark features of osteoporosis. Understanding the operation of these signaling pathways and their modulation under pathological conditions is essential for identifying effective therapeutic strategies. For instance, BMP2 has been approved by the FDA for clinical applications, such as long bone fracture repair and spinal fusion, owing to its ability to promote osteoblast differentiation [41]. Although its potential in treating osteoporosis has been explored, long-term use of BMP2 has been associated with increased osteoclast activity and excessive bone resorption. Furthermore, clinical reports have documented adverse effects, including vertebral osteolysis, hematoma, and seroma formation, limiting its suitability for osteoporosis treatment [42]. Despite these limitations, the BMP signaling pathway remains a valuable target for further research. A deeper understanding of its mechanisms may uncover new therapeutic avenues to modulate bone formation and resorption more precisely, paving the way for safer and more effective treatments for osteoporosis.

### Role of oxidative stress, ferroptosis, and inflammation

Oxidative stress is a condition characterized by an imbalance between oxidative and antioxidant systems, favoring oxidation in the body. This imbalance is primarily driven by ROS and is typically mitigated by antioxidant enzymes. When the redox balance is disturbed, oxidative stress can develop, significantly contributing to aging and the progression of various diseases, including osteoporosis [43]. Excess free radicals, particularly ROS, adversely affect bone health by disrupting bone remodeling processes. ROS impair osteoblast function by inhibiting the expression of essential osteogenic transcription factors such as Runx2 and Osx, which are critical for bone formation. Concurrently, ROS promote osteoclastogenesis by upregulating markers like c-Fos, NFATc1, and tartrate-resistant acid phosphatase (TRAP), thereby enhancing bone resorption [44]. Moreover, oxidative stress influences bone metabolism through interactions with glutathione (GSH) and the induction of ferroptosis. GSH, a vital antioxidant, is crucial for maintaining cellular redox homeostasis. However, excessive ROS can deplete GSH levels, diminishing its protective capacity and triggering ferroptosis, an iron-dependent form of regulated cell death [45]. This process not only exacerbates oxidative damage but also disrupts bone homeostasis. Collectively, these findings underscore the complex interplay between oxidative stress, ferroptosis, and bone remodeling, elucidating their combined contributions to skeletal degeneration and osteoporosis.

Hydrogen peroxide (H_2_O_2_) is frequently employed in cellular models to induce oxidative stress by mimicking ROS effects. In studies related to bone health, exposure to H_2_O_2_ has been shown to hinder the osteogenic differentiation of BMMSCs by downregulating key osteogenic markers such as ALP and Col1, while also inhibiting mineralization. Furthermore, H_2_O_2_ disrupts the stability of the Wnt/β-catenin signaling pathway, which is essential for osteoblast function and bone formation [46]. In addition to suppressing osteogenesis, H_2_O_2_ promotes BMMSC senescence and enhances adipogenic differentiation, both negatively impacting bone regeneration [47]. Previous studies indicate that exogenous H_2_O_2_ inhibits osteoblast differentiation and induces apoptosis by damaging the mitochondrial antioxidant defense system [48, 49]. Additionally, H_2_O_2_ impairs autophagy by inhibiting the PI3K/AKT/mTOR pathway and induces pyroptosis in osteoblasts through the activation of caspase-1 expression [50]. Beyond its effects on osteoblasts, H_2_O_2_ facilitates osteoclastogenesis by promoting the differentiation of BMMs into osteoclasts via macrophage polarization. During this process, H_2_O_2_ activates the NF-κB and MAPK signaling pathways, further enhancing osteoclast activity and bone resorption [51]. Collectively, these findings illustrate the multifaceted role of H_2_O_2_ in disrupting bone homeostasis by impairing osteoblast function, enhancing osteoclastogenesis, and interfering with critical signaling pathways involved in bone remodeling.

Ferroptosis is a distinct form of non-apoptotic programmed cell death characterized by iron-dependent lipid peroxidation (LPO). It is implicated in various metabolic disorders and conditions associated with disrupted cellular homeostasis. Unlike apoptosis and autophagy, ferroptosis follows a separate regulatory pathway. A defining feature of ferroptosis is its close association with ROS, with mitochondria and ROS-producing enzymes such as NADPH oxidase 4 (NOX4) identified as significant sources of ROS in bone tissue [52]. Recent research has demonstrated that mitochondria and NOX4 play integral roles in regulating ferroptotic pathways [53]. Within mitochondria, ROS generation occurs during electron transfer to molecular oxygen, primarily from the electron transport chain (ETC) or the tricarboxylic acid (TCA) cycle. Over 90% of electrons transferred to O_2_ generate superoxide (O_2_^−^), designating mitochondria as a critical site of oxidative stress. The TCA cycle and the ETC contribute to ferroptosis by serving as primary sources of intracellular lipid peroxide production, further amplified by NOX4 activity, which produces ROS using NADPH as a substrate. Upon activation, NOX4 promotes the accumulation of lipid peroxides, thereby triggering ferroptosis, as demonstrated in glioma cells. As lipid peroxides accumulate, they initiate extensive LPO, generating several cytotoxic byproducts, including malondialdehyde (MDA), lipid hydroperoxides (LOOH), and 4-hydroxynonenal (4-HNE) [45, 54]. These interconnected processes highlight the central role of mitochondrial metabolism and NOX4 activity in driving ferroptotic cell death through oxidative lipid damage.

To counteract pro-ferroptotic signals, cells depend on antioxidant defense systems, particularly glutathione peroxidase 4 (GPX4), which neutralizes lipid peroxides and prevents their accumulation to cytotoxic levels. When antioxidant capacity is compromised, either through GSH depletion or excessive ROS production, cells become increasingly susceptible to ferroptotic death. Factors such as cystine depletion, which limits GSH synthesis, and ROS overproduction exacerbate this vulnerability by accelerating LPO [55]. Due to its central regulatory role, GPX4 is commonly utilized as a molecular marker to assess ferroptosis in various disease models.

Recent studies have identified ferroptosis as a key regulator of inflammatory responses in various pathological conditions. This iron-dependent form of cell death contributes to a self-perpetuating inflammatory loop through several mechanisms. Notably, ferroptosis promotes the release of pro-inflammatory cytokines, including interleukin-1β (IL-1β), interleukin-6 (IL-6), and tumor necrosis factor-alpha (TNF-α). In turn, TNF-α activates the NF-κB signaling pathway, amplifying inflammation and oxidative stress by promoting leukocyte recruitment and enhancing ROS production [56]. Moreover, IL-6 and TNF-α are recognized as key regulators of ferritin synthesis, further reinforcing their role in ferroptosis-related pathways [57]. Concurrently, ferroptosis is increasingly acknowledged as a critical contributor to bone metabolism disorders. In particular, ferroptosis in osteoblasts has been shown to significantly impact bone loss and the progression of osteoporosis.

The dynamic regulation of osteoclastogenic and anti-osteoclastogenic cytokines is crucial for maintaining bone homeostasis. During macrophage polarization, various inflammatory mediators influence osteoclastogenesis by exerting either pro-inflammatory or anti-inflammatory effects, thus impacting bone resorption and contributing to the progression of osteoporosis. TNF-α promotes osteoclast differentiation by upregulating RANK-associated pro-inflammatory genes through the activation and nuclear translocation of NF-κB. This process disrupts the RANK–RANKL signaling axis and enhances osteoclast activity. Additionally, IL-1β and IL-6 drive osteoclast differentiation and maturation via RANKL-independent pathways, ultimately leading to increased bone resorption [58].

### Hormonal influences

Osteoporosis is primarily driven by age-related physiological changes and increased bone resorption due to a deficiency in sex hormones. Among these hormones, estrogen insufficiency is particularly significant in both men and women; however, its effects are more pronounced in women due to the abrupt decline in estrogen levels that occurs after menopause. Reduced levels of estradiol (E2) in postmenopausal women lead to an increase in the production of pro-inflammatory cytokines by circulating monocytes, which can differentiate into tissue macrophages. These macrophages contribute to local inflammation and the activation of osteoclasts. In contrast, IL-4 possesses anti-inflammatory and bone-protective properties by regulating osteoclast differentiation [59]. Collectively, these cytokines enhance RANKL expression, activating osteoclasts and accelerating bone resorption, which contributes to significant trabecular bone loss [60].

Estrogen regulates bone metabolism by binding to ERs, including ERα, ERβ, and G protein-coupled receptor 30 (GPR30), which are differentially expressed in osteoblasts and osteoclasts. A reduction in the expression or activity of these receptors is closely associated with bone loss, as evidenced in postmenopausal women and ovariectomized (OVX) mouse models. Specifically, the deletion of ERα in osteoblast lineage cells has been shown to decrease cortical BMD, while targeted deletion of ERα in osteoclast lineage cells enhances osteoclastogenesis and bone resorption, ultimately leading to a deterioration of trabecular bone microarchitecture [61]. Furthermore, global depletion of ERs in mice, either through ERα knockout (ERα–/–) or the double knockout of ERα and ERβ (ERα Δ–/–), impairs bone remodeling in both cortical and trabecular regions, resulting in reduced bone mass in both sexes [62]. Collectively, these findings underscore the critical roles of estrogenic signaling and ER expression in maintaining skeletal integrity. Consequently, modulating estrogen pathways and targeting ERs represent promising therapeutic strategies for preventing osteoporosis and reducing the risk of associated bone fractures.

## Key phenolic metabolites involved in bone health

**Figure 2.**
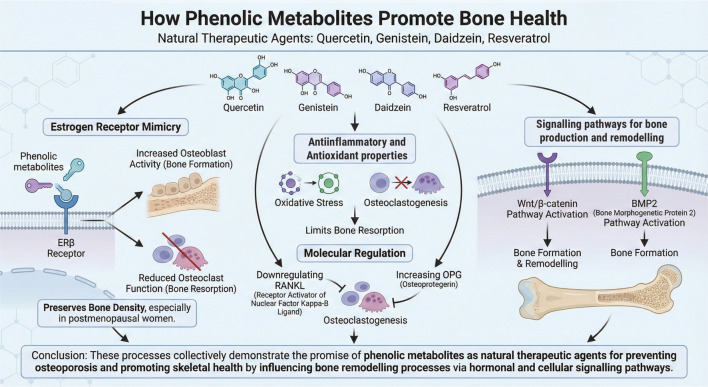
**Molecular mechanisms of phenolic-derived secondary metabolites in regulating bone remodelling via hormonal and cellular signaling pathways.** Structures of genistein, daidzein, quercetin, resveratrol, and 17β-oestradiol are shown with pathways indicating enhanced osteoblastogenesis (activation of Wnt/β-catenin, BMP2/Smad and Nrf2/HO-1; inhibition of p38 MAPK; ↑Runx2/osteogenic markers/OPG) and reduced osteoclastogenesis (↓RANKL and pro-inflammatory cytokines; Nrf2/HO-1 activation; inhibition of the ferroptosis/NOX4 axis). Abbreviations: BMP2: Bone morphogenetic protein 2; Nrf2: Nuclear factor erythroid 2-related factor 2; HO1: Heme oxgenase-1; MAPK: Mitogen-activated protein kinase; ALP: Alkaline phosphatase; RANKL: Receptor activator of nuclear factor-κB ligand; NOX4: Nicotinamide adenine dinucleotide phosphate (NADPH) oxidase 4.

Phenolic metabolites, such as quercetin, genistein, daidzein, and resveratrol, promote bone health through various mechanisms. They mimic estrogen and bind to ERs, particularly ERβ, to enhance osteoblast activity and inhibit osteoclast function. This process aids in the preservation of bone density, especially in postmenopausal women. Additionally, these metabolites exhibit anti-inflammatory and antioxidant properties that reduce oxidative stress and inhibit osteoclastogenesis, thereby limiting bone resorption. They further minimize osteoclastogenesis by downregulating RANKL and increasing the expression of OPG. Moreover, these metabolites influence the Wnt/β-catenin and BMP2 pathways, which are essential for bone production and remodeling, illustrating their potential as natural therapeutic agents for preventing osteoporosis and promoting skeletal health through hormonal and cellular signaling pathways (Figure 2).

### Flavonoids

Flavonoids, a subclass of polyphenols, are recognized for their distinct chemical structures and diverse biological activities. Also known as bioflavonoids or plant flavonoids, they are abundantly found in dietary plants, including fruits, vegetables, legumes, and tea, in both free and bound forms. Structurally, flavonoids are characterized by a common backbone consisting of two phenolic rings (designated as A and B rings) connected by a three-carbon bridge, forming a central heterocyclic ring [63]. Numerous flavonoids have been shown to influence key components of bone-related signaling pathways, particularly the Wnt and BMP pathways [64].

Among them, quercetin (Que), also known as 3,3′,4′,5,7-pentahydroxyflavone, is a widely studied flavonoid belonging to the flavonol subclass. Que is prevalent in a variety of fruits and vegetables and is recognized for its potential therapeutic effects in bone disorders such as osteoporosis [65]. In osteoporotic conditions, Que has been reported to enhance the gene expression of crucial osteogenic transcription factors, including Runx2 and Osx, thereby promoting bone formation [66]. Additionally, Que has demonstrated protective effects against oxidative stress in ferric ammonium citrate-treated MC3T3-E1 cells by activating the Nrf2/HO-1 signaling pathway [67]. Furthermore, it promotes osteogenic activity in osteoblasts by upregulating the expression of Cbfα1/Runx2 and bone sialoprotein (BSP) genes in rat osteoblast-like ROS cells [68]. Elevated concentrations of Que-glucoside, particularly at 10 and 100 µM, further enhanced key osteogenic markers, including ALP activity, mineralization, and the production of OCN, Runx2, BMP2, and Col1 [69].

Que facilitates the osteogenic differentiation of MC3T3 E1 cells by increasing β-catenin protein levels and activating the Wnt/β-catenin pathway [70]. It further enhances the proliferation and osteogenic differentiation of BMMSCs by modulating the H19/miR-625-5p axis. Que upregulates H19 while suppressing miR-625-5p, leading to increased β-catenin accumulation and downstream Wnt signaling [71]. In ovariectomized rats, Que administered at medium to high doses raised OPG expression and lowered RANKL levels in femoral tissue, suppressing bone resorption, preventing osteoporosis, and improving femoral biomechanical properties [9]. Additionally, Que (2–5 µM) significantly reduces TNF-α and IL-6 production in LPS-stimulated RAW 264.7 macrophages [72]. Tsai et al. (2021) reported that Que inhibits M1 macrophage and microglial polarization, markedly decreasing the expression of pro-inflammatory markers IL-6, TNF-α, and IL-1β. By suppressing TNF-α and IL-1β, Que attenuates osteoclast activation and mitigates bone destruction [73].

Beyond its established antioxidant and anti-inflammatory properties, emerging evidence suggests that Que may exert biological effects via estrogen-mediated pathways. Structurally similar to endogenous estrogens, Que is classified as a phytoestrogen and has been shown to bind to ERs, particularly ERβ, albeit with lower affinity compared to 17β-estradiol [74]. Through this interaction, Que may mimic or modulate estrogen signaling in bone tissue, thereby contributing to the regulation of bone remodeling processes. As a member of the flavonoid class, Que shares structural features with estrogen and has demonstrated estrogen-like activity in various biological systems, including those related to breast cancer. Notably, findings from Pang et al. (2018) showed that treatment with the ER antagonist ICI182780 significantly reduced the expression of osteogenic transcription factors Runx2 and Osx, as well as osteopontin (OPN), in BMMSCs. This inhibition was observed in cells treated with both quercetin and E2, indicating that the osteogenic effects of Que are at least partially mediated through ER signaling [75]. These results support the hypothesis that estrogen signaling plays a critical role in Que-induced osteogenesis, highlighting its potential relevance in managing postmenopausal bone loss.

Multiple *in vivo* studies have demonstrated Que’s protective role against bone loss by enhancing BMD, improving bone microarchitecture and strength, promoting bone growth, reducing bone resorption markers, and increasing bone formation markers [76, 77]. Yurteri et al. [78] further reported that Que also contributed to bone strengthening during both the early and late phases of fracture healing.

### Isoflavones

Genistein (C_15_H_10_O_5_), also known as 4′,5,7-trihydroxyisoflavone, is a naturally occurring isoflavone and secondary metabolite primarily found in leguminous plants, as well as in seeds, fruits, and vegetables. In these natural sources, it predominantly exists in glycosylated forms, which are hydrolyzed into the biologically active aglycone during digestion or food processing. As a phytoestrogen, genistein structurally and functionally resembles mammalian 17β-oestradiol, featuring a characteristic diphenolic structure that serves as a scaffold for the development of synthetic estrogens. Genistein is recognized for its diverse biological and pharmacological properties, including antioxidant, anti-inflammatory, anticancer, antidiabetic, neuroprotective, hepatoprotective, and bone-protective effects [79]. Its mechanisms of action involve interactions with multiple cellular signaling pathways. Notably, genistein binds to ERs (ERα and ERβ), with a higher affinity for ERβ, thereby modulating estrogen-dependent gene expression [80]. Additionally, genistein activates or inhibits several key intracellular signaling cascades, including the Wnt/β-catenin pathway, PI3K/Akt pathway, NF-κB signaling, and p38 MAPK pathways. Through these pathways, it exerts diverse effects on cell proliferation, differentiation, survival, and inflammation [81]. These pleiotropic actions underscore its therapeutic potential in various chronic and degenerative diseases, including osteoporosis.

Genistein has been shown to enhance ALP activity in a time-dependent manner, along with upregulating osteogenesis-related markers such as OCN and Runx2 in rat osteoblasts by increasing the expression of ERs alpha (ERα) [82]. Similarly, genistein promotes the expression of genes involved in osteoblast differentiation and stimulates mineralization in MC3T3-E1 cells by upregulating ERα and activating the MAPK/NF-κB/AP-1 signaling pathway [83]. Furthermore, genistein significantly enhances the expression of Wnt10b by more than 60-fold in primary osteoblasts, indicating a strong proliferative effect, along with substantial increases in BMP6 and Runx2 levels (over 50-fold). In osteocyte cell lines, genistein modulates the Wnt/β-catenin signaling pathway by upregulating Wnt10b, promoting β-catenin nuclear translocation, and reducing sclerostin, a key inhibitor of the Wnt pathway produced by osteocytes [84]. These findings suggest that genistein promotes bone formation by activating β-catenin signaling, leading to increased expression of osteogenic markers such as BMP6 and Runx2 in osteoblasts.

Genistein reduces the production of ROS by activating the NRF2/HO-1 signaling pathway, suppressing NADPH oxidase 1 (NOX1), and preventing disruption of the mitochondrial ETC in RANKL-treated RAW264.7 cells [85]. In these pre-osteoclastic RAW264.7 murine macrophage cells, genistein effectively inhibits RANKL-induced osteoclast differentiation and activity. Moreover, genistein has demonstrated the potential to act synergistically with the bisphosphonate alendronate, enhancing its inhibitory effects on osteoclast formation [86]. This suggests a promising therapeutic approach for preventing and treating osteoporosis. Consistently, genistein treatment has been linked to the regulation of the OPG/RANKL system and improved BMD in OVX rats. Additionally, genistein has exhibited synergistic effects with silicon in counteracting OVX-induced bone loss and BMD reduction, as evidenced by a significant decrease in RANKL expression and an increase in OPG levels in serum and bone tissue [87].

Daidzein, chemically known as 7-hydroxy-3-(4-hydroxyphenyl)-4H-1-benzopyran-4-one, is a naturally occurring phytoestrogen classified as a nonsteroidal estrogen. It is commonly found in soy-based food products, including soy infant formula, soy flour, textured soy protein, soy protein isolates, tofu, and miso [88]. In bone, daidzein has been shown to enhance the phosphorylation of Smad1/5/8 and increase Osx protein expression, thereby activating BMP signaling and stimulating the production of collagen type I, Runx2, and ALP [89]. Additionally, daidzein elevates protein and mRNA levels of OPG, reduces the expression of RANKL and the inflammatory cytokine IL-6, and activates the classical estrogen response element (ERE) pathway [90]. Collectively, these effects promote the proliferation and differentiation of osteoblasts.

An *in vivo* study investigated the combined effects of daidzein and calcium on preserving bone mass and biomechanical strength in OVX mice. The results indicated that daidzein was metabolized into equol in all mice and did not induce uterotrophic effects [91]. Estrogen deficiency is known to increase bone turnover and accelerate bone loss, ultimately heightening fracture risk. Beyond its estrogenic properties, equol, an isoflavone metabolite of daidzein, has been shown to inhibit bone loss following ovariectomy [92]. O-desmethylangolensin (O-DMA) and equol are the primary metabolites of daidzein formed in the gastrointestinal tract, with variations in intestinal microflora accounting for differing effects on bone metabolism. Notably, equol supplementation was found to maintain BMD in the proximal, distal, and whole femur, whereas O-DMA did not produce similar outcomes [93]. Furthermore, several studies indicated that resistant starch, a form of starch that supports the conversion of daidzein into equol, can enhance urinary equol excretion, tibial BMD, and the systemic availability of daidzein [94].

### Stilbene

Resveratrol (RSV), or 3,5,4′-trihydroxystilbene, is a naturally occurring polyphenolic stilbene compound primarily found in the skins of red grapes, as well as in mulberries, peanuts, and pine trees. Through its diverse bioactivity, RSV interacts with several intracellular targets, including receptors, enzymes, signaling molecules, antioxidant enzymes, and transcription factors [95]. These interactions contribute to RSV’s ability to inhibit NF-κB and RANKL-mediated osteoclastogenesis, suppress oxidative stress and inflammation, while simultaneously promoting osteogenesis by enhancing the differentiation of MSCs into osteoblasts. The mechanisms underlying these effects involve various signaling pathways and regulatory proteins, such as the Wnt/β-catenin, PI3K/AKT, and BMP2 pathways. Importantly, RSV is recognized as one of the most potent activators of SIRT1, a key regulator that stimulates osteoblast activity while concurrently inhibiting osteoclast formation [96].

Additionally, RSV is classified as a phytoestrogen due to its ability to act as an ER agonist, exhibiting varying degrees of activity across different cell types. At a concentration of 0.1 µM, RSV has been shown to enhance ER expression in a time-dependent manner, with maximal expression observed after 48 h, without altering the expression of ER isoforms [97]. Molecular docking analysis revealed that RSV interacts with the catalytic amino acid triad within the ER binding pocket, demonstrating favorable binding energy toward both ER isoforms. Notably, the binding mode of RSV closely resembles that of the natural hormone 17β-oestradiol [98]. These findings suggest that RSV’s estrogen-mimicking activity contributes to its therapeutic potential as a bone anabolic agent, particularly in the context of postmenopausal osteoporosis treatment. In this context, *in vivo* studies have demonstrated that RSV significantly increases BMD in ovariectomized rats. This bone-protective effect is primarily attributed to RSV’s ability to promote osteogenesis by downregulating pro-inflammatory cytokines, including TNF-α, IL-1β, IL-6, and IL-1, as well as by reducing the RANKL/OPG ratio, thereby inhibiting osteoclastogenesis and enhancing bone formation [99]. Beyond postmenopausal osteoporosis, which has a distinct pathophysiology primarily driven by estrogen deficiency, several *in vivo* studies have demonstrated the bone-protective effects of RSV in other forms of osteoporosis, such as senile and disuse-induced models. These findings suggest that RSV may exert broader therapeutic potential by targeting common mechanisms of bone loss, including oxidative stress, inflammation, and impaired bone remodeling, irrespective of the underlying cause.

## Clinical evidence supporting the anti-osteoporotic potential of phenolic compounds

BMD serves as a static measure of bone composition, reflecting the long-term history of bone health. However, significant changes in BMD may take several years to become detectable. In contrast, bone turnover markers (BTMs) present in circulation provide insight into more immediate changes in bone remodeling activity, potentially serving as early predictors of future changes in bone density and strength. Bone formation markers, such as OCN and procollagen type I N-terminal propeptide (PINP), secreted by osteoblasts, indicate bone-forming activity. Conversely, bone resorption markers, such as the C-terminal telopeptide of type I collagen (CTX), are released during bone degradation and reflect osteoclast-mediated resorption. The balance between bone formation and resorption is tightly regulated, with estrogen playing a central role in maintaining this equilibrium by inhibiting excessive bone breakdown [100].

Estrogen also modulates the immune response by downregulating the production of pro-inflammatory cytokines such as interleukin (IL)-1, IL-6, and TNF-α. These cytokines, known to stimulate osteoclast activity, can be further upregulated by systemic inflammatory markers such as C-reactive protein (CRP). In postmenopausal women, estrogen deficiency leads to increased production of these cytokines, resulting in heightened osteoclast activity and accelerated bone resorption [60]. Consequently, this shift contributes to reduced BMD over time, a process detectable earlier through changes in BTMs before manifesting in traditional BMD measurements. Table 1 summarizes the pharmacological effects of phenolic-derived secondary metabolites with anti-osteoporotic action, supported by clinical findings. Studies were retrieved from the Scopus and WOS databases using the search string (“phenol” OR “phenolic compounds” OR “polyphenol” OR “isoflavones” OR “genistein” OR “daidzein” OR “quercetin” OR “stilbene” OR “lignan” OR “resveratrol”) AND (“bone” OR “osteoporosis”) AND (clinical). Studies published from database inception through September 2025 were considered, focusing on the effects of phenolic compounds on bone health, their mechanisms of action, and clinical findings related to osteoporosis.

**Table 1:** Pharmacological effects of phenolic-derived secondary metabolites with anti-osteoporotic action

**Models**	**Treatment, dose, period**	**Outcome**	**Reference**
Postmenopausal women	Quercetin, 500 mg, 3 months	Quercetin significantly increased levels of osteocalcin, PINP, and CTX, while simultaneously reducing levels of IL-6 and TNF-α when compared to the placebo control. Notably, levels of CRP and BMD remained unchanged.	[101]
Postmenopausal women	Dasatinib 100 mg/day + Quercetin 1000 mg/day, administered intermittently for 2 consecutive days each month	Combination treatment with dasatinib and quercetin significantly increased P1NP levels at both 2 and 4 weeks. The skeletal response to this combination was particularly evident in women with a high burden of senescent cells, as indicated by the upper tertile of T cell p16 expression, and was associated with enhanced radial bone mineral density at 20 weeks.	[102]
Postmenopausal women	Isoflavones, 500 mg, 12 weeks	Isoflavones significantly increased levels of osteocalcin and PINP compared to the placebo control group.	[103]
Premenopausal women	Isoflavones, 136.6 mg, 5 days per week for 2 years	Isoflavones increased calcium levels and BMD.	[104]
Premenopausal women	Isoflavones, 136.6 mg, 5 days per week for 2 years	Isoflavones have been shown to enhance BMD. Specifically, genistein excretion resulted in a reduction of whole-body BMD at low-normal serum calcium levels, while it increased BMD at elevated calcium levels.	[105]
Postmenopausal women	Genistein, 54 mg, 24 months	Genistein has been shown to enhance BMD in postmenopausal women, including those with osteoporosis.	[106]
Postmenopausal women	GeniVida™ Bone Blend (GBB) comprises genistein (30 mg/day), vitamin D3 (800 IU/day), vitamin K1 (150 µg/day), and polyunsaturated fatty acids, administered over a period of six months	GBB increased ALP, PINP and BMD.	[107]
Postmenopausal women	Ipriflavone, 200 mg 3 times per day, calcium, 500 mg, 4 years	Ipriflavone did not significantly affect BMD or biochemical markers of bone resorption, specifically urinary hydroxyproline corrected for creatinine.	[108]
Postmenopausal women	Resveratrol, 75 mg (twice daily), 2 years	Resveratrol enhanced ALP and osteocalcin while decreasing CTX and aminoterminal proCNP (NTproCNP) levels compared to the placebo control.	[116]
Postmenopausal women	Fermented soy (200 mg containing 10 mg of equol and 25 mg of RSV), 12 months	The combination of equol and resveratrol increased BMD and ALP levels while decreasing DPD levels. In contrast, TRACP-5b levels remained unchanged.	[117]

Abbreviations: PINP: Procollagen type I N-terminal propeptide; CRP: C-reactive protein**;** CTX: C-terminal telopeptide of type I collagen; BMD: Bone mineral density; ALP: Alkaline phosphatase; NTproCNP: Aminoterminal proC-type natriuretic peptide; TRACP-5b: Tartrate-resistant acid phosphatase 5b; DPD: Deoxypyridinoline.

### Quercetin

A study by Bailly et al. (2025) investigated the effects of 90-day Que supplementation on BTMs, inflammation, BMD, body composition, and physical function in 33 healthy postmenopausal women. In this double-blind, placebo-controlled trial, participants were randomized to receive either 500 mg of Que or a placebo (methylcellulose) daily. Pre- and post-testing included BTMs (OCN, PINP, CTX), inflammatory markers (IL-6, TNF-α, CRP), BMD, body composition, and functional tests (timed up and go, handgrip strength). Compared to the placebo group, the Que group exhibited increased levels of OCN, PINP, and CTX, along with reduced levels of IL-6 and TNF-α. No significant changes were observed in CRP, BMD, body composition, or physical function. These findings suggest that Que may help regulate bone turnover by promoting formation and reducing inflammation. However, the concurrent rise in CTX raises questions about its long-term protective effects on bone, warranting further research in postmenopausal populations [101].

Farr et al. (2024) conducted a phase 2 randomized controlled trial to assess the senolytic effects of dasatinib and quercetin (D + Q) on bone health in 60 postmenopausal women aged 62–88 years. Participants in this 20-week open-label study received either a control or the senolytic combination of dasatinib (100 mg/day) and quercetin (1000 mg/day), administered intermittently for two consecutive days each month. D + Q therapy significantly lowered blood CTX levels, indicating decreased bone resorption, and resulted in temporary elevations in the bone formation marker P1NP at weeks 2 and 4, although this effect did not persist for the full 20 weeks. No significant adverse effects were reported. Exploratory analyses revealed that subjects with a higher senescent cell load (based on T-cell p16 mRNA expression) exhibited better skeletal responses, including increased P1NP, lower CTX at 2 weeks, and higher radial BMD at 20 weeks. These findings indicate that, while D + Q did not substantially affect bone resorption overall, individual responses may vary depending on baseline senescent cell burden, necessitating further investigation [102].

The study by Bailly et al. (2025) provides a nuanced perspective on the potential effects of Que supplementation at a dosage of 500 mg/day for 90 days on bone health in postmenopausal women. The observed modulation of BTMs, specifically an increase in bone formation markers such as OCN and P1NP, along with a reduction in pro-inflammatory cytokines (IL-6, TNF-α) and CRP, suggests that quercetin may exert osteo-regulatory and anti-inflammatory effects relevant to maintaining skeletal health in this population [101]. However, the concurrent increase in the bone resorption marker CTX, despite remaining within clinically acceptable limits, raises concerns regarding the overall balance of bone remodeling. If the elevation in resorption persists, it could potentially offset the benefits of enhanced bone formation, thereby limiting the protective effects of Que on bone mass and structural integrity. In summary, while the results indicated that Que may positively influence bone metabolism and systemic inflammation, the concurrent increase in bone resorption necessitates cautious interpretation. These findings underscore the need for longer-duration, larger-scale clinical trials to determine whether Que supplementation can provide a clinically significant protective effect against postmenopausal bone loss, osteoporosis, and fracture risk.

### Isoflavones

Clinical evidence more consistently supports the bone-protective effects of isoflavones. In a recent double-blind, placebo-controlled randomized trial (RCT), 100 postmenopausal women were assigned to receive soy extract nutraceuticals over 12 weeks. Results indicated that participants in the soy extract group experienced significant improvements in BTMs, including PINP and OCN, compared to the placebo group, suggesting a beneficial impact on bone health [103]. In another study involving 197 healthy premenopausal women, 99 participants were randomized to receive soy isoflavones (136.6 mg aglycone equivalents), while 98 received a placebo, administered five days per week for up to two years. BMD, serum calcium, and urinary excretion of daidzein and genistein were measured before and during treatment [104]. Among 129 adherent participants, isoflavone exposure, assessed by urinary genistein excretion (GE), interacted with serum calcium in influencing whole-body BMD, although no effects were observed at the hip or spine. Genistein decreased whole-body BMD at low-normal serum calcium levels but increased it at higher calcium levels. Comparing high vs low GE, changes in whole-body BMD ranged from +0.033 to −0.113 g/cm^2^ at serum calcium levels of 10 and 8.15 mg/dL, respectively. These associations were not detected in intention-to-treat analysis, highlighting variability in isoflavone metabolism. Overall, isoflavones may influence calcium homeostasis by mobilizing calcium from bones, particularly under conditions of low serum calcium [105].

Genistein, a soy isoflavone, has garnered attention for its potential to prevent bone loss during menopause, supported by evidence from animal models and clinical trials. A post-hoc analysis of a randomized, double-blind, placebo-controlled trial evaluated the effects of genistein supplementation in postmenopausal women with low BMD. Participants were assigned to receive either calcium and vitamin D3 alone (control) or a combination of calcium, vitamin D3, and genistein. At baseline, a similar proportion of women in both groups were classified as osteoporotic. Over the 24-month intervention, women receiving genistein demonstrated a significant increase in femoral neck BMD, while BMD declined in the control group. Concurrently, the prevalence of osteoporosis decreased in the genistein group but remained unchanged in the control group. These findings suggest that genistein may provide additional benefits in managing osteopenia and osteoporosis in postmenopausal women, beyond standard calcium and vitamin D3 supplementation [106]. Complementing these findings, another double-blind pilot study examined the effects of a genistein-based bone blend (GBB) containing genistein, vitamin D3, vitamin K1, and polyunsaturated fatty acids in early postmenopausal women. Over six months, participants receiving GBB maintained their BMD at critical sites, such as the femoral neck, while those on calcium alone experienced significant decreases in BMD. The GBB was well tolerated, with no significant difference in adverse events compared to placebo. This study reinforced the potential bone-protective effects of genistein, particularly when combined with other supportive nutrients, suggesting it may reduce fracture risk [107]. Collectively, these studies highlight genistein’s promising role as a non-pharmaceutical option for osteoporosis prevention, although larger, long-term clinical trials are necessary to confirm its efficacy and safety.

Before 2002, numerous clinical trials investigating isoflavones, particularly synthetic derivatives such as ipriflavone, administered relatively high doses of approximately 600 mg/day to evaluate their efficacy in preventing bone loss. This approach was driven by limited early evidence, leading researchers to use larger doses. Although ipriflavone proved effective in preventing bone loss and increasing bone mass, subsequent studies raised concerns regarding potential adverse effects associated with high-dose isoflavone intake, including liver toxicity, immune system suppression, and an increased risk of lymphocytopenia. In response to these safety concerns, recent studies have shifted toward employing lower doses of isoflavones, typically ranging from 35 to 150 mg/day, to balance efficacy with a reduced risk of side effects [108].

Despite this trend, some commercially available supplements still contain isoflavone amounts that exceed the typical E2-equivalent doses used in postmenopausal women, raising significant safety considerations. This is particularly relevant for individuals with pre-existing health conditions or increased sensitivity to hormone-related effects. Accurate dosing necessitates calculating E2-equivalent concentrations based on isoflavone aglycone equivalents, as various isoflavone derivatives differ in their capacity to activate ERs ERα and ERβ. For instance, a high-dose study administering 600 mg of ipriflavone aglycone equivalent (IAE) corresponded to E2 equivalents of 66 mg for ERα and 18 mg for ERβ. Conversely, a lower-dose study with 18 mg IAE, primarily comprising daidzein and genistein, resulted in substantially lower E2 equivalents for both receptors [109, 110]. These findings underscore the necessity for precise dosing to ensure the safe and effective use of isoflavones, particularly for long-term supplementation.

### Stilbene

RSV has been shown to enhance systemic and cerebral circulation, likely through the activation of endothelial ERs. A pilot study by Chow et al. (2014) evaluated the effects of RSV intervention on systemic sex steroid hormones in postmenopausal women with a higher body mass index. Although the treatment did not significantly alter serum levels of E2, estrone, or testosterone, it did lead to an approximately 10% increase in sex hormone-binding globulin (SHBG) concentrations, which may enhance estrogen metabolism [111]. Supporting these clinical findings, experimental studies in ovariectomized rodent models, designed to mimic postmenopausal osteoporosis resulting from estrogen deficiency, suggested that RSV exerts a protective effect on bone health [96, 99]. Collectively, these results indicate that resveratrol may influence bone metabolism, at least in part, by modulating estrogen-related pathways, thereby affecting hormone availability in humans and directly protecting bone tissue in animal models.

Wong et al. (2020) conducted the RESHAW trial, a 24-month randomized, double-blind, placebo-controlled crossover study, to assess the effects of RSV supplementation on cognition, cerebrovascular function, bone health, cardiometabolic markers, and overall well-being in postmenopausal women. After 12 months of RSV treatment compared to placebo, participants exhibited improvements in bone density at the lumbar spine and femoral neck, alongside a significant reduction in a key marker of bone resorption. These changes coincided with enhanced bone strength scores and a decreased 10-year risk of major fractures, with the most notable benefits observed in women with poorer baseline bone health. Furthermore, improvements in femoral neck bone strength were associated with increased blood flow to the area. A subgroup analysis revealed that the bone-protective effects of RSV were more pronounced in women supplemented with vitamin D and calcium [112]. These effects were accompanied by a reduction in C-terminal telopeptide type I collagen levels, a marker of bone resorption [113].

C-type natriuretic peptide (CNP) is a paracrine growth factor essential for endochondral bone growth in mammals, including humans. Animal studies have indicated that CNP signaling promotes osteoblast proliferation and osteoclast activity [114, 115], although its role in bone remodeling within the mature skeleton remains unclear. Utilizing plasma samples from the RESHAW trial, which investigated RSV supplementation in postmenopausal women with mild osteopenia, researchers examined changes in plasma aminoterminal proCNP (NT-proCNP) alongside BTMs of formation (OCN and ALP) and resorption (CTX) over a two-year period involving 125 subjects. Participants received either a placebo or RSV in the first year, with treatments switched in the second year. Results revealed no overall correlation between NT-proCNP and BTMs. However, NT-proCNP levels significantly declined during the first year in both groups, with a more pronounced decrease observed following RSV treatment compared to the placebo group. ALP levels increased following RSV, while other markers remained unchanged. Notably, NT-proCNP was inversely associated with BMD at the lumbar spine following RSV treatment, implying a potential role for CNP in bone remodeling during periods of increasing bone density [116]. This study provides initial evidence that CNP is modulated during bone health interventions in postmenopausal women, indicating a need for further research.

The recent investigation by Corbi et al. (2020) aimed to assess the effects of combined equol (an isoflavone metabolite of daidzein) and RSV supplementation on bone turnover biomarkers in postmenopausal women. Sixty healthy postmenopausal participants were randomly assigned to receive either 200 mg of fermented soy containing 10 mg of equol and 25 mg of RSV or a placebo for a duration of 12 months. Measurements of whole-body BMD and key BTMs, including deoxypyridinoline (DPD), TRACP-5b, OCN, and bone-specific ALP (BAP), were taken at baseline and after the intervention. After 12 months, significant improvements were observed in DPD, OCN, and BAP levels in the supplemented group compared to the placebo group, while TRACP-5b levels remained unchanged. Within-group analyses revealed statistically significant changes from baseline in DPD, OCN, and BAP concentrations. Additionally, whole-body BMD increased significantly in the treatment group compared to the placebo [117]. These findings suggest that supplementation with equol and RSV can positively influence BTMs and enhance BMD, presenting a promising strategy to mitigate age-related bone loss in postmenopausal women.

## Recent research on phenolic bioavailability

Following gastrointestinal absorption, phenolic compounds are transported into enterocytes and subsequently into hepatocytes, where they undergo extensive intracellular metabolism. Within these cells, phenolic glycosides are subjected to enzymatic hydrolysis by intracellular β-glucosidases, particularly broad-specificity cytosolic β-glucosidase, an enzyme abundantly expressed in the liver, kidney, and small intestine [118]. The rate and extent of deglycosylation are significantly influenced by the structure of the aglycone, as well as the position, type, and steric hindrance of glycosidic linkages. Mono-glycosylated flavonoids, such as quercetin-4′-glucoside, naringenin-7-glucoside, apigenin-7-glucoside, genistein-7-glucoside, and daidzein-7-glucoside, are efficiently hydrolyzed by intestinal and hepatic β-glucosidases. Among these, genistein-7-glucoside exhibits a higher enzymatic affinity and a faster rate of deglycosylation compared to quercetin-4′-glucoside. Conversely, more complex glycosides like quercetin-3,4′-diglucoside, quercetin-3-glucoside, kaempferol-3-glucoside, quercetin-3-rhamnoglucoside, and naringenin-7-rhamnoglucoside show low enzymatic susceptibility and often remain unmetabolized, thereby limiting their bioavailability [119].

Within hepatocytes, the parent phenolics and phase II metabolites generated in enterocytes undergo further biotransformation, including glucuronidation, deglucuronidation, sulfation, methylation (via catechol-O-methyltransferase, COMT), glycine conjugation, and phase I metabolic reactions [120]. Quercetin 7-O-glucuronide and 3-O-glucuronide, as major metabolites formed in enterocytes, are delivered to the liver, where they may simultaneously undergo methylation by COMT and deglucuronidation via β-glucuronidase [121].

During oral administration, phenolic compounds undergo significant first-pass metabolism, involving extensive enzymatic modification and efflux across intestinal and hepatic barriers. This metabolic process substantially reduces the plasma concentration and bioactivity of phenolics before they reach systemic circulation and exert therapeutic effects. Although some phenolics may be reabsorbed through enterohepatic and enteroenteric recirculation, the majority, including both unabsorbed and conjugated forms, are ultimately transported to the colon [122]. There, due to limited digestibility, poor permeability, extensive phase II metabolism, and active efflux, a considerable proportion of phenolics is subjected to microbial catabolism by the colonic microbiota.

Absorption limitations are further exacerbated by the glycosylated nature of many flavonoids, especially those existing as β-glycosides, which are generally not absorbable in their native form. In contrast, aglycones can more readily diffuse across the intestinal epithelium [123]. To address these challenges, nanocarrier systems, due to their high surface area-to-volume ratio and enhanced mucosal interaction, can improve drug–membrane interactions, facilitating more efficient absorption. Advanced delivery technologies such as nanoencapsulation, prodrug design, and liposome-based formulations have been developed to enable controlled gastrointestinal release, enhance aqueous solubility, and ultimately improve the systemic bioavailability and therapeutic efficacy of phenolic compounds.

### Nanoencapsulation

Polymeric nanoparticles and naturally derived nanocarriers are among the most effective and industrially scalable platforms for the protection and targeted delivery of phenolic compounds. These systems facilitate nanoencapsulation, wherein phenolics are embedded into sub-micron solid particles, enhancing their chemical stability, aqueous solubility, and bioavailability [124]. Depending on the formulation method and structural configuration, nanoparticles are typically classified as nanospheres or nanocapsules. Nanospheres are matrix systems in which the bioactive compound is uniformly distributed throughout the polymeric matrix. In contrast, nanocapsules feature a core–shell architecture, with the active compound enclosed in a liquid core surrounded by a polymeric membrane. This structural difference significantly influences release kinetics, protection efficiency, and gastrointestinal absorption [125].

Regarding absorption challenges, many flavonoids exhibit poor intestinal uptake due to their presence as β-glycosides, which are not readily absorbed compared to their aglycone counterparts [119]. Nanocarriers present a promising strategy to overcome this limitation, as their high dispersion properties enhance interaction with the intestinal epithelium, promoting drug transport and uptake. Moreover, encapsulation within edible nanocarriers, such as cyclodextrins, biopolymer-based particles, and lipid-based systems, provides additional benefits, including biocompatibility, controlled release, and protection from environmental degradation, making them suitable for applications in food, pharmaceuticals, and nutraceuticals [126].

Que, a lipophilic bioactive compound, has been effectively encapsulated in poly(lactic acid) (PLA) nanoparticles using the solvent evaporation technique, achieving an encapsulation efficiency of 96.7% and a drug loading of 19.4%. Antioxidant activity assays confirmed that the functional properties of quercetin were preserved following nanoencapsulation. The resulting formulation exhibited a biphasic release profile, characterized by an initial burst release followed by a prolonged sustained release phase [127]. The combination of high encapsulation efficiency, nanoscale particle size, and controlled release kinetics positions quercetin-loaded PLA nanoparticles as a promising platform for the development of nanomedicine-based delivery systems. Kang et al. (2023) investigated the enhancement of antioxidant activity and solubility of Que and isoquercetin (IQue) through nanoencapsulation and gel incorporation. Nanoparticles were prepared via ionic gelation of chitooligosaccharide and poly-γ-glutamic acid. Antioxidant activity increased 2.5-fold for Que and 3.5-fold for IQue after nanoencapsulation. Incorporating these nanoparticles into gelatin gels (G-Que and G-I Que NPs) further improved antioxidant stability [128].

A novel nanocombination formulation of the flavonoids Que and curcumin was developed for anti-osteoporotic therapy, utilizing polylactic-co-glycolic acid (PLGA) as the encapsulating polymer matrix. Findings suggested that co-delivery of Que and curcumin using PLGA exerted a synergistic protective effect against OVX-induced bone degradation, supporting its potential as a nanotherapeutic strategy for osteoporosis management. The co-encapsulated PLGA nanoparticles exhibited a nanoscale size range of 110–135 nm with a homogeneous particle distribution, indicating favorable physicochemical properties for systemic delivery. High encapsulation efficiency and drug loading capacity confirmed the formulation’s suitability for dual-drug delivery [129]. *In vitro* cytotoxicity assays confirmed that the nanoparticles were cytocompatible with bone-related cells. Meanwhile, *in vivo* studies using OVX rats, a common model for postmenopausal osteoporosis and age-related skeletal muscle changes, showed that the co-loaded nanoparticles effectively prevented trabecular bone loss and significantly improved BMD and bone microarchitecture [130]. These effects were linked to the activation of key signaling pathways, including the Wnt and BMP pathways, as well as the miR-206/Connexin43 axis, which promotes osteogenesis [131]. Additionally, in a drill-hole bone defect model in mice, localized delivery of selenium nanoparticle-based Que (Qu-SeNPs) via hydrogel significantly accelerated bone healing. Overall, these well-characterized Qu-SeNPs support bone remodeling, and when embedded in hydrogels, may enhance cellular uptake and bioavailability, offering promising potential for advanced orthopedic and regenerative treatments targeting bone loss and defects [131].

Fang et al. (2022) developed a PEGylated cyclodextrin-based nanoplatform (PCP) for the localized delivery of RSV-loaded nanomicelles (RSV-NM) targeting inflammatory osteolysis. Given the critical roles of excessive osteoclast activity and ROS in the pathogenesis of bone loss, coupled with the ROS-scavenging capacity of RSV, the authors engineered an ROS-responsive delivery system by incorporating phenylboronic acid ester moieties into the nanocarrier structure. The resulting formulation displayed enhanced solubility, improved physicochemical stability, and excellent biocompatibility compared to free RSV. *In vitro* assays confirmed that RSV-NM effectively inhibited osteoclastogenesis, indicating its therapeutic potential for managing inflammation-induced bone resorption [132]. Additionally, Peng et al. (2023) developed a novel nanoplatform, RSV@DTPF, designed for targeted and controlled delivery of RSV to macrophages in a ROS-responsive manner. The platform featured a folate-modified surface that enhances cellular uptake by binding to folate receptors on macrophages, and a thioketal linker that breaks under high ROS conditions to release RSV. *In vitro* studies demonstrated the nanoparticles’ ability to scavenge ROS, rebalance the M1/M2 macrophage ratio in an LPS-induced inflammatory environment, promote osteoblast differentiation, and inhibit osteoclast maturation. A co-culture system further validated the immunomodulatory role in bone remodeling. *In vivo*, RSV@DTPF significantly promoted osteogenesis and alveolar bone regeneration in a periodontal defect model using ovariectomized rats, confirmed through imaging and histological analysis. The study highlighted the potential of RSV@DTPF to modulate the immune microenvironment and enhance bone regeneration in osteoporosis, with promising applications in broader biomedical contexts related to oxidative stress and redox imbalance [133].

### Prodrug

Prodrugs are pharmacologically inactive derivatives of active compounds, strategically designed through chemical modification to enhance pharmacokinetic and physicochemical properties. These molecules undergo *in vivo* transformation, typically via enzymatic or chemical hydrolysis, into their pharmacologically active form. A primary objective of prodrug development is to improve solubility, stability, membrane permeability, and bioavailability, thereby enhancing therapeutic efficacy. Notably, approximately 7% of all approved drugs are classified as prodrugs [134].

For instance, RSV prodrugs effectively attenuated colon inflammation in a murine model of dextran sulfate sodium (DSS)-induced colitis. Protection from rapid phase II metabolism and excretion enabled improved colonic targeting and enhanced local anti-inflammatory activity [135]. To address RSV’s rapid metabolic clearance, Mattarei et al. (2015) developed a prodrug by conjugating isoleucine to RSV via an N-monosubstituted carbamate ester, resulting in improved solubility, metabolic stability, and sustained release. However, the formulation exhibited low oral absorption in rats, likely due to the high hydrophilicity introduced by three ionizable carboxylic acid groups [136]. Another RSV prodrug, 3,5-triethylsilyl-4′-(6′′-octanoylglucopyranosyl) resveratrol, demonstrated superior therapeutic efficacy in murine models of Huntington’s disease and multiple sclerosis, highlighting the versatility of prodrug design in addressing various pathological conditions [137].

### Liposome

Liposomes are spherical vesicular systems composed of one or more concentric phospholipid bilayers that enclose an aqueous core. They can encapsulate hydrophilic compounds within their aqueous interior and hydrophobic agents within the lipid bilayer. Due to their biocompatibility, structural versatility, and high encapsulation efficiency, liposomes are widely utilized as drug delivery vehicles. A significant limitation in the systemic administration of liposomal formulations is their rapid clearance by the reticuloendothelial system (RES), primarily through phagocytic uptake [138]. To address this issue, surface modification with hydrophilic polymers such as polyethylene glycol (PEG), a process known as PEGylation, is regarded as the gold standard. PEGylation enhances the pharmacokinetic profile by reducing opsonization, prolonging systemic circulation time, and enabling passive or active targeting through ligand-receptor interactions [139].

Liposomes spontaneously assemble when amphiphilic phospholipids are dispersed in an aqueous medium, driven by thermodynamic forces that favor bilayer formation. Their physicochemical properties, including size, surface charge, and lamellarity, can be finely tuned during formulation to optimize drug loading and release characteristics. Liposomal systems exhibit minimal immunogenicity, low intrinsic cytotoxicity, and the ability to modulate biodistribution profiles, making them highly favorable for therapeutic applications [138]. Depending on their size and the number of bilayers, liposomes can range from small unilamellar vesicles (SUVs, ∼20–100 nm) to large multilamellar vesicles (MLVs, >500 nm). When the diameter of the vesicles is less than approximately 200 nm, they are referred to as “nanoliposomes.” These nanoscale liposomes are particularly advantageous for targeted drug delivery due to their enhanced permeability and retention (EPR) effect in tumor tissues [140].

Senescence of BMMSCs is a critical contributor to the pathogenesis of osteoporosis. The senolytic combination of dasatinib and quercetin (DQ) has been investigated for its ability to mitigate bone loss by selectively eliminating senescent cells. In a study by Li et al., alendronate-functionalized liposomes encapsulating DQ (Aln-Lipo-DQ) were developed to target senescence-associated osteoporosis, particularly that induced by chemotherapy or radiotherapy. Alendronate, a bisphosphonate with a high affinity for hydroxyapatite, facilitates the targeted delivery of the liposomal formulation to bone tissue, specifically the femur and tibiae. Treatment with Aln-Lipo-DQ effectively reduced the burden of senescent cells in bone and significantly increased the bone volume fraction from 5.05% to 11.95% in a chemotherapy-induced osteoporosis mouse model. In a radiotherapy-induced model, Aln-Lipo-DQ treatment resulted in a 2.91-fold increase in bone volume fraction compared to untreated controls. These findings underscore the potential of bone-targeted senolytic therapy in addressing cancer therapy-related and age-associated osteoporotic conditions by selectively depleting senescent cells from skeletal tissues [141].

Isoquercitrin (IQ), a glycosylated derivative of quercetin, is metabolized to its aglycone form following oral administration, thereby exerting similar pharmacological activities. However, its clinical application has been limited due to poor stability and bioavailability [142]. To address these challenges, Sheng et al. (2024) developed IQ-loaded PEGylated long-circulating liposomes (IQ-Lips) using the thin-film hydration method. The resulting formulation exhibited nanoscale particle size and improved physicochemical properties. Oral administration of IQ-Lips significantly enhanced the aqueous solubility, systemic bioavailability, and circulation time of IQ. Importantly, in an osteoporosis model, treatment with IQ-Lips resulted in improved bone mass and a reduction in oxidative stress markers, indicating its therapeutic potential in managing osteoporosis-associated bone loss and oxidative damage [143].

## Conclusion and future perspectives

Osteoporosis, characterized by decreasing bone mass and microarchitectural degradation, is closely related to oxidative stress and chronic inflammation, both of which can be regulated by phenolic substances. Preclinical studies demonstrate that phenolics protect bones by activating Nrf2-mediated antioxidant defenses, inhibiting NF-κB signaling, modulating the RANKL/OPG balance, and stimulating osteoblast differentiation through the Runx2 and Wnt/β-catenin pathways. These mechanisms are supported by experimental data showing that specific flavonoids (e.g., quercetin and genistein) and stilbenes (e.g., resveratrol) promote osteoblastogenesis, suppress osteoclast activity, and maintain bone remodeling equilibrium in the context of aging, estrogen deficiency, or pharmaceutical stress.

Translationally, these molecular findings are partially consistent with human clinical results, which indicate that phenolic supplementation modestly improves BTMs and inflammatory profiles. However, clinical evidence remains limited due to small cohort sizes, short intervention periods, and demographic variability, which restrict substantial conclusions regarding BMD or fracture outcomes. While formulation innovations, including liposomal encapsulation and nanoparticle-based delivery methods, are being explored to improve bioavailability, many findings to date remain in the preclinical proof-of-concept stage. Overall, phenolic secondary metabolites present promising multi-targeted therapies for osteoporosis prevention, but larger, mechanistically guided clinical trials are necessary to establish their efficacy and therapeutic potential.

## Supplemental data

**Graphical abstract**

**Figure:**